# Screening of *Chlamydia trachomatis* and *Waddlia chondrophila* Antibodies in Women with Tubal Factor Infertility

**DOI:** 10.3390/microorganisms8060918

**Published:** 2020-06-17

**Authors:** Wies T.M. van Dooremalen, Stephan P. Verweij, Janneke E. den Hartog, Carole Kebbi-Beghdadi, Sander Ouburg, Gilbert Greub, Servaas A. Morré, Anne Ammerdorffer

**Affiliations:** 1 Laboratory of Immunogenetics, Department of Medical Microbiology and Infection Control, Amsterdam UMC, Location AMC, 1105 AZ Amsterdam, The Netherlands; wiesvandooremalen@outlook.com (W.T.M.v.D.); stephanverweij@msn.com (S.P.V.); s.ouburg@amsterdamumc.nl (S.O.); sa.morre@amsterdamumc.nl (S.A.M.); 2 TubaScan LTD., 1098 XG Amsterdam, The Netherlands; 3 Department of Obstetrics & Gynaecology, GROW School of Oncology and Developmental Biology, Maastricht University Medical Center+, 6229 ER Maastricht, The Netherlands; janneke.denhartog@mumc.nl; 4 Institute of Microbiology, University Hospital Center and University of Lausanne, 1011 Lausanne, Switzerland; Carole.Kebbi-Beghdadi@chuv.ch (C.K.-B.); gilbert.greub@chuv.ch (G.G.); 5 Institute for Public Health Genomics, Department of Genetics and Cell Biology, Research Institute GROW, Faculty of Health, Medicine & Life Sciences, University of Maastricht, 6229 ER Maastricht, The Netherlands

**Keywords:** *Waddlia chondrophila*, *Chlamydia trachomatis*, tubal factor infertility, female reproductive health, serology

## Abstract

*Waddlia chondrophila* is an emerging intracellular pathogen belonging to the order of *Chlamydiales*, and was previously associated with adverse pregnancy outcomes, as well as tubal factor infertility (TFI). In this study, we investigate the link between both *W. chondrophila* and *Chlamydia trachomatis* IgG seropositivity and TFI. Antibodies against both bacteria were measured in 890 serum samples of women visiting a fertility clinic. After a hysterosalpingography and/or laparoscopy, they were classified as either TFI-negative (TFI−) or TFI-positive (TFI+). The total seroprevalence was 13.4% for *C. trachomatis* and 38.8% for *W. chondrophila*. *C. trachomatis* antibodies were present significantly more often in the TFI+ group than in the TFI− group, while for *W. chondrophila* no difference could be observed. In conclusion, our study confirms the association between *C. trachomatis* seropositivity and TFI, but no association was found between *W. chondrophila* seropositivity and TFI. The high percentage of *W. chondrophila* seropositivity in all women attending a fertility clinic does, however, demonstrate the need for further research on this *Chlamydia*-like bacterium and its possible role in infertility.

## 1. Introduction

While most women will get pregnant within one year of trying, 10–15% of couples worldwide are having difficulties to conceive [1]. Not only is this a large emotional burden for these couples, the financial costs for treatment, such as in vitro fertilization (IVF), are also increasing in our society. To increase treatment efficiency, it is important to know what the causes of infertility are and how they can be diagnosed early.

Part of the infertility cases are due to female factors, such as ovulatory dysfunction and tubal factor infertility (TFI) [2,3]. Among the causes of TFI, including endometriosis and surgical traumas, are bacterial infections of the lower and upper genital tract [4]. The well-studied intracellular bacterium *Chlamydia trachomatis* is one of the most important pathogens involved in these infertility-related infections. *C. trachomatis* is the most common sexually transmitted bacterial infection (STI), with more than 1.1 million female cases reported in the USA in 2018 and around 60,000 cases in both males and females in the Netherlands in 2018 [5,6]. *C. trachomatis* can effectively be treated with antibiotics such as azithromycin and doxycycline [7]. However, in more than 70% of cases, a *C. trachomatis* infection is asymptomatic and thus goes unnoticed and untreated [8]. When left untreated, the infection may lead to the development of pelvic inflammatory disease, which may also run asymptomatic, and/or persistent infections, which are major causes of TFI [5,9].

Because of the important role of *C. trachomatis* in the development of TFI, a serological screening test was introduced in the Netherlands in 1998 [9]. The *Chlamydia*-IgG-antibody test (CAT) detects the presence of *C. trachomatis* IgG antibodies in serum, and is positive in up to 80% of women with TFI [9,10]. Upon screening, women are classified as having a high risk (CAT-positive) or low risk (CAT-negative) of TFI. Because not all CAT-positive women develop tubal damage, additional diagnostics are performed to confirm or exclude TFI. Women will undergo tubal testing by hysterosalpingography and/or laparoscopy, which is a more specific but invasive and costly procedure. The results of these diagnostic tests define the presence and grade of TFI and determine the further course of action, e.g., referral for IVF in case of TFI, and expectant management or mild ovarian stimulation with intra-uterine insemination in women without tubal abnormalities.

Several studies identified *Waddlia chondrophila*, an emerging intracellular bacterium belonging to the order of *Chlamydiales*, to be associated with adverse pregnancy outcomes and infertility, such as TFI [10,11,12,13,14,15]. It is worth noting that *W. chondrophila* efficiently replicates in human endometrial cells [16] and ovine trophoblast cells [17], where its highly immunogenic proteins [18] might induce significant inflammation, possibly leading to local scarring and TFI and/or miscarriages [19,20]. *W. chondrophila* does not seem to spread through sexual contact but infection might rather occur via consumption of milk and uncooked meat or via contact with animals [11]. A study performed by Baud et al. showed a *W. chondrophila* seroprevalence of 33% in a group of English women suffering from recurrent miscarriages, as compared to 7.1% in women with uneventful pregnancies [14]. They later confirmed these findings in Swiss women and visualized the presence of *W. chondrophila* in the placentas of three infected women using immunohistochemistry, of which two had a miscarriage [12,21]. In 2015, our group published a study in which serum samples from women with and without TFI were tested for *W. chondrophila* antibodies [10]. This revealed a high prevalence in the total study population (520 infertile women) for *W. chondrophila* (45.5%). High titers of *W. chondrophila* antibodies were associated with severe TFI, independent of *C. trachomatis* titers. Currently, there are only limited studies in which the seroprevalence of *W. chondrophila* is studied among the general population [22,23,24]. One study by Baud et al. investigated the seroprevalence among 482 asymptomatic Swiss men (average age of 20 years) and observed a positive *W. chondrophila* IgG in 8.3% of the subjects [22]. No publications are available on the *W. chondrophila* seroprevalence in a large group of healthy individuals.

In this current study, we investigate *C. trachomatis* and *W. chondrophila* seroprevalence in a cohort of 890 women with infertility, to establish a possible link between seropositivity to these bacteria and TFI.

## 2. Materials and Methods

### 2.1. Study Population

In total, 891 Dutch women participated in the study. They had either attended the fertility clinic of the Maastricht University Medical Center + (MUMC+, *n* = 315) between 2005–2017 or the University Medical Center Groningen (UMCG, *n* = 576) between 2007–2013 because of infertility, i.e., not having conceived after at least one year of unprotected intercourse. The UMCG cohort was previously used by Verweij et al. [10]. In the study period, clinical procedures were similar in both centers. As part of the fertility work-up, blood was drawn in all women for CAT, and spare serum was cryopreserved. High risk patients for TFI (CAT-positives) were offered laparoscopy with methylene blue dye testing, unless severe male factor infertility (requiring IVF/ICSI) was diagnosed. Low risk patients for TFI (CAT-negatives) underwent hysterosalpingography, and in case of abnormal findings, laparoscopy was offered.

### 2.2. TFI Definition

According to their laparoscopic (LS) and/or hysterosalpingography (HSG) scores, women were classified as TFI-negative (TFI−) or TFI-positive (TFI+). Within the TFI+ group, a distinction for severe TFI (sTFI) is made. TFI− is defined by HSG 0 and/or LS 0 (no or few adhesions and open tubes), while TFI+ is defined as no or few adhesions and one proximally or distally occluded tube (LS 1), or extensive adhesions and/or no open tubes (LS 2) (Table 1). The latter condition is defined as sTFI. In case that HSG = 1, TFI is only confirmed by an additional LS 1 or 2. Patients with a non-conclusive TFI status were excluded from the study (*n* = 1, resulting in 314 women from MUMC+).

### 2.3. Ethical Approval

The act “Medical Research Involving Human Subjects” (WMO, Dutch Law) states that anonymous spare human materials and data may be used for research purposes after patients have been informed about this possible use and they have had the opportunity to object. All samples used in the study are anonymous spare human materials, and none of the participants objected to the use of the material for future research purposes. The usage of the samples has been reviewed by the local medical ethics review committee from the MUMC+ (year 2017, project code MUMC+ 2017–0232) and UMCG (year 2016, project code UMCG METc2016.309).

### 2.4. The Chlamydia-IgG-Antibody Test (CAT)

In MUMC+ and UMCG, CAT has been measured in routine care during the fertility work-up by pELISA Medac (Medac Diagnostika mbH, Hamburg, Germany). The following outcomes are considered: <22 AU/mL is negative, 22–28 AU/mL is grey zone, >28 AU/mL is positive. A CAT value in the grey zone is classified as a negative CAT.

### 2.5. Detection of Antibodies against W. chondrophila

*W. chondrophila* antibodies in the serum samples were measured by ELISA, as described previously [10,25]. The ELISA uses enriched outer membrane proteins isolated from purified elementary bodies of *W. chondrophila* as antigens. Optical densities (OD) were measured using the ELISA Multiskan ascent reader (Thermo scientific, Zurich, Switzerland) at 492 nm, against 650 nm as reference. All experiments were performed in duplicate. Control sera were included as reference to calculate ROC curves and cut-off levels for positivity, negativity, and grey zone. Details concerning the control sera and defining the cut-off levels can be found in the paper of Lienard et al., in which they describe the development of the *W. chondrophila*-specific ELISA [25]. Samples from the MUMC+ cohort were measured in three subsets, cut-off values for seropositivity were set at 0.395, 0.359 and 0.737. Seropositivity cut-off levels for the UMCG cohort were set at 0.164. The highest 10% of *W. chondrophila*-positive OD value ratios were selected as a high *W. chondrophila*. These ratios were determined by dividing the OD values by their respective cut-off value.

### 2.6. Statistics

Descriptive statistics were performed on the cohorts. Data were presented as number of individuals (% of total individuals). Categorical data were compared between groups using Fisher’s Exact test. Risk factors were described as Odds Ratio (OR) with a 95% confidence interval (CI). *p*-values < 0.05 were considered statistically significant, 0.05 < *p* < 0.1 was considered a statistical trend. Analyses were performed using GraphPad Prism (version 8.2.1., GraphPad Software, CA, US).

## 3. Results

From all included patients, the CAT status and HSG and/or LS results were available. Patient characteristics and performed procedures of the two cohorts are described in Table 2. The average age of the women at the time of the CAT measurement was 31.5 years, and in both cohorts around 13% were CAT-positive. 676 (76.0%) patients received a hysterosalpingography, of whom 60 also had a laparoscopy. In total, 274 (30.8%) of the patients had a laparoscopy. In addition, the TFI status for the MUMC+ and the UMCG cohort were comparable with, respectively, 87.9% and 88.4% having no TFI, and, respectively, 12.1% and 11.6% of the patients being diagnosed with TFI.

The seroprevalence of *C. trachomatis* within the total cohort was 13.4% compared to a 38.8% seroprevalence of *W. chondrophila* (*p* < 0.0001; OR: 4.1; 95% CI: 3.24–5.17). An overview of the seroprevalence of *C. trachomatis* and *W. chondrophila* in individuals with and without TFI is summarized in Table 3.

*C. trachomatis* antibodies were present significantly more often in the TFI+ compared to the TFI− group, respectively, 41.9% versus 9.6% (*p* < 0.0001; OR: 6.8; 95% CI: 4.28–10.76). In the sTFI group, the prevalence of *C. trachomatis* (43.9%) was similar to that of the total TFI+ group (41.9%). In contrast to *C. trachomatis*, the prevalence of *W. chondrophila* antibodies was similar in both the TFI+ and TFI− group (*p*: 0.457; OR: 0.8; 95% CI: 0.55–1.30), with 39.2% testing positive in the TFI− group and 35.2% and 31.8% in the TFI+ and sTFI group, respectively.

Subsequently, we investigated whether serum samples that were highly positive for *W. chondrophila* were associated with TFI. From the 345 positive *W. chondrophila* samples, the highest 10% were considered highly positive (WC-H). Overall, these samples corresponded with 3.9% of the total cohort. From the 35 individuals in the WC-H group, 6 were TFI-positive and 29 were TFI-negative, corresponding with 5.7% of the TFI+ group and 3.7% of the TFI− group (*p*: 0.43; OR: 1.5; 95% CI: 0.61–3.48). Furthermore, no significant difference was observed between high seropositivity for *W. chondrophila* in women with sTFI versus the TFI− group (*p*: 0.52; OR: 1.5; 95% CI: 0.53–4.08).

Women with antibodies against both *C. trachomatis* and *W. chondrophila* were more likely to have TFI than women with either or none of these antibodies (*p*: 0.0016; OR: 3.4; 95% CI: 1.71–6.82), although this effect was not stronger than the effect of *C. trachomatis* alone (*p* < 0.0001; OR: 6.8; 95% CI: 4.28–10.76). The same trend was seen for women who were positive for *C. trachomatis* and highly positive for *W. chondrophila*, although this was not significant (*p*: 0.109; OR: 5.1; 95% CI: 0.97–25.0). 

Within the TFI+ group, we further distinguished women with either proximal or distal occlusion of at least one tube, or a combination of both proximal and distal occlusion (Table 4). In total, 49 women were diagnosed with at least one distally occluded tube, of which more women had a positive serology for *C. trachomatis* (51.0%) than for *W. chondrophila* (30.6%) IgG antibodies (*p*: 0.064; OR: 2.4; 95% CI: 1.00–5.49). Among the 22 women with proximal occlusions, 45.5% had a positive *W. chondrophila* serology versus 31.8% of *C. trachomatis* (*p*: 0.54; OR: 1.8; 95% CI: 0.57–6.38). Four women had bilaterally occluded tubes, of whom three were positive for *C. trachomatis* and none for *W. chondrophila.* In the sTFI subgroup (Table 5), the *C. trachomatis* prevalence (60.7%) remained higher than *W. chondrophila* (32.1%) for women with at least one distally occluded tube (*p*: 0.059; OR: 3.3; 95% CI: 1.15–8.88). From the eight women with proximal occlusions, the seropositivity was 25% for both bacteria.

## 4. Discussion

In the current study, we investigated the seroprevalence of *C. trachomatis* and *W. chondrophila* in women with or without TFI. Hereto, we made use of a large cohort consisting of 890 women who attended a fertility clinic, either at the MUMC+ or the UMCG in The Netherlands. As the women were included at two different locations, several characteristics of both groups were first evaluated. In both groups, the average age at the time of CAT and the percentage of women receiving hysterosalpingography and/or laparoscopy were comparable. In addition, the TFI scoring (HSG 0 or 1, LS code 0, 1 or 2) was discussed among the two gynecologists and performed in a similar way. The CAT has been measured using pELISA Medac, this ELISA is one of the more reliable *C. trachomatis* ELISAs, as shown in a study comparing 5 different ELISAs [26].

Although the UMCG cohort was used in a similar study by Verweij et al. [10], we decided to combine the MUMC+ and UMCG cohort for several reasons. First of all, using a larger number of individuals in our study results in more reliable data and statistics. Especially because the TFI+ and sTFI groups include more individuals, and these groups are the central subject for our study. Secondly, new definitions are used to define TFI. Because we want to examine all TFI cases and not only *C. trachomatis*-associated TFI, the current study does not exclude women based on their medical background, and women with i.e., endometriosis or tubal surgery are also included. Moreover, the distinction is no longer made between proximal and distal occlusions, but the emphasis is now to what extent abnormalities can influence the chance of a spontaneous pregnancy (i.e., present in case of one-sided and absent in case of two-sided abnormalities). As we investigate the role of *W. chondrophila* on the development of TFI in general, we decided to follow the new definitions.

In concordance with other studies, a significantly higher *C. trachomatis* seroprevalence is observed in the TFI+ group (41.9%) compared to the TFI− group (9.6%) [9,10,27,28]. This high percentage of *C. trachomatis* antibodies in women with TFI is similar to the 45% found by Price et al. [27] and 41.7% by Verweij et al. [10]. When comparing the TFI+ versus sTFI group, we observed no difference in the *C. trachomatis* seropositivity. This is in contrast with the previous study by Verweij et al., where a seroprevalence of 41.7% was observed in the TFI+ group versus 80.0% in the sTFI group [10]. This can be explained by the fact that the study from Verweij et al. only contained 10 individuals with severe TFI, versus 66 in our study, and that the definitions for severe TFI differ between the two studies.

In contrast to *C. trachomatis*, no association between a positive *W. chondrophila* serology and TFI could be observed. There was no significant difference in the seroprevalence of *W. chondrophila* between the TFI+ (35.2%) and TFI− group (39.2%). In addition, the sTFI group also showed a similar seroprevalence (31.8%). In the study from Verweij et al., the percentage of *W. chondrophila* seropositivity in TFI+ women was comparable to the percentage of *C. trachomatis* seropositivity (both 41.7%) [10]. In our current study, the seropositivity for *W. chondrophila* in TFI+ individuals is 35.2%, which is slightly lower than the 41.9% for *C. trachomatis*.

The current study and the previous study by Verweij et al. show that the *W. chondrophila* seroprevalence was similar in both the TFI+ and TFI− group. Although these two studies are the only ones investigating the role of *W. chondrophila* in the development of TFI, other studies were published that investigated the role of *W. chondrophila* in other female reproductive health problems (reviewed in [19,29]). In two studies performed by Baud et al., the role of *W. chondrophila* was examined in women with (recurrent) miscarriages or uneventful pregnancies [12,14]. In those studies, the control groups had a *W. chondrophila* seroprevalence between 7.1% and 14.6%, which is much lower than the 39.2% and 46.0% found in our studies. However, it has to be mentioned that the serological diagnostic techniques were different, using immunofluorescence versus ELISA, respectively. Moreover, the TFI− women in our studies did visit the fertility clinic, indicating that there might be other clinical reasons for their infertility that might be associated with high *W. chondrophila* seropositivity.

We could not confirm the association that Verweij et al. found between a high *W. chondrophila* seropositivity and (severe) TFI. A possible explanation for this is the change in TFI definitions (new definitions Table 1), leading to a larger percentage of women with severe TFI in this study than in the previous study (7.4% versus 2.1%).

One of the causes of proximal and distal tubal damage is inflammation of the fallopian tubes [4], partly due to bacterial infections. It has been suggested in the past that distal occlusions are more associated with TFI as compared to proximal occlusions [30]. In our study, we have observed a high prevalence of *C. trachomatis* antibodies (51.0%), specifically in women with distally occluded tubes. This is in accordance with findings from previous studies [31]. For *W. chondrophila*, no difference in antibody prevalence was observed between women with either proximal or distal occlusions.

In conclusion, this study confirms the association between *C. trachomatis* seropositivity and TFI, but no association was found between the presence of *W. chondrophila* IgG antibodies and TFI. We do however observe a high seroprevalence for *W. chondrophila* in all the women who attended a fertility clinic, suggesting that there might be a role for this bacterium in infertility other than tubal pathology.

It would be interesting for future studies to investigate the association of *W. chondrophila* with other female reproductive health issues as well, such as (early and late) miscarriage, ectopic pregnancy and pelvic inflammatory disease (PID), and to investigate the antibody presence against other *Chlamydia*-like bacteria such as *Parachlamydia acanthamoebae* or *Simkania negevensis* in women with TFI. At the moment, there are only limited studies on the association between *Chlamydia*-like bacteria and these clinical outcomes [11,32]. It can be valuable to look at differences in local mucosal antibody responses between women with and without TFI, and to have a closer look at cellular responses against *W. chondrophila* antigens.

However, the fact that previous reports show an association, and that infections with the related *C. trachomatis* are associated as well, gives reasons to continue the research. Moreover, further research will provide more answers to women and their families who suffer from miscarriages or other infertility issues with unidentified causes.

## Figures and Tables

**Table 1:** Overview of new hysterosalpingography (HSG), Laparoscopy (LS) and TFI definitions.

**HSG**	**Definition**
0	Considered as not affecting fertility, no consequences
1	Considered as affecting fertility, further diagnostics indicated
**LS**	**Definition**
0	No or few adhesions and open tubes (fertility not compromised)
1	No or few adhesions and one proximally or distally occluded tube (fertility possibly compromised)
2	Extensive adhesions and/or no open tubes (infertility)
9	Not conclusive
**TFI**	**Definition**
Negative (TFI−)	HSG 0 and/or LS 0
Positive (TFI+)	LS 1 and/or LS 2
Severe (sTFI)	LS 2 only

**Table 2:** Characteristics of the MUMC+ and UMCG cohorts combined and individually.

Cohort	N	CAT+ (%)	Age at Time of CAT	HSG (%)	LS (%)	TFI− (%)	TFI+ ^1^ (%)	sTFI (%)
Combined	890	119 (13.4)	31.5	676 (76.0)	274 (30.8)	785 (88.2)	105 (11.8)	66 (7.4)
MUMC+	314	45 (14.3)	31.4	228 (72.6)	99 (31.5)	276 (87.9)	38 (12.1)	28 (8.9)
UMCG	576	74 (12.8)	31.6	448 (77.8)	175 (30.4)	509 (88.4)	67 (11.6)	38 (6.6)

Hysterosalpingography (HSG), laparoscopy (LS) and tubal factor infertility (TFI) definitions are described in Table 1. TFI− = HSG 0 and/or LS 0, TFI+ = LS 1 and 2, sTFI = LS 2. ^1^ Includes patients with sTFI.

**Table 3:** *C. trachomatis* and *W. chondrophila* prevalence within the total cohort (*n* = 890).

	N	CT ^1^ (%)	WC ^2^ (%)	WC-H (%)	CT and WC (%)	CT and WC-H (%)
Total	890	119 (13.4)	345 (38.8)	35 (3.9)	41 (4.6)	5 (0.6)
TFI−	785	75 (9.6)	308 (39.2)	29 (3.7)	29 (3.7)	3 (0.4)
TFI+ ^3^	105	44 (41.9)	37 (35.2)	6 (5.7)	12 (11.4)	2 (1.9)
sTFI	66	29 (43.9)	21 (31.8)	4 (6.1)	8 (12.1)	1 (1.5)

CT: *C. trachomatis.* WT: *W. chondrophila.* ^1^ Includes patients co-infected with WC. ^2^ Includes patients with WC-H and patients co-infected with CT. ^3^ Includes patients with sTFI.

**Table 4:** Tubal occlusions in TFI+ individuals.

TFI+ (*n* = 105)	N	CT ^1^ (%)	WC ^2^ (%)	CT and WC (%)
≥1 distal occlusion	49	25 (51.0)	15 (30.6)	8 (16.3)
≥1 proximal occlusion	22	7 (31.8)	10 (45.5)	2 (9.1)
Distal + proximal occlusions	4	3 (75.0)	0	0
No extensive adhesions	26	9 (34.6)	11 (42.3)	2 (7.7)
Unknown (no tubal testing performed)	4	0	1 (25.0)	0

^1^ Includes patients co-infected with WC. ^2^ Includes patients with WC-H and patients co-infected with CT.

**Table 5:** Tubal occlusions in sTFI individuals.

sTFI (*n* = 66)	N	CT ^1^ (%)	WC ^2^ (%)	CT and WC (%)
≥1 distal occlusion	28	17 (60.7)	9 (32.1)	6 (21.4)
≥1 proximal occlusion	8	2 (25.0)	2 (25.0)	0
Distal + proximal occlusions	4	3 (75.0)	0	0
No extensive adhesions	22	7 (31.8)	9 (40.9)	2 (9.1)
Unknown (no tubal testing performed)	4	0	1 (25.0)	0

^1^ Includes patients co-infected with WC. ^2^ Includes patients with WC-H and patients co-infected with CT.
